# Midwives’ integration of post abortion manual vacuum aspiration in the Democratic Republic of Congo: a mixed methods case study & positive deviance assessment

**DOI:** 10.1186/s12913-020-05997-7

**Published:** 2020-12-10

**Authors:** Kirsty M. Bourret, Sylvie Larocque, Amélie Hien, Carol Hogue, Kalum Muray, Aurélie Thethe Lukusa, Abel Minani Ngabo

**Affiliations:** 1 School of Midwifery, Faculty of Health Sciences, Laurentian University, 935 Ramsey Lake road, Sudbury, ON Canada; 2 School of Nursing, Faculty of Health Sciences, Laurentian University, Sudbury, Canada; 3 Department of French studies, Laurentian University, Sudbury, Canada; 4 Jules & Uldeen Terry Professor Emerita of Maternal and Child Health, Professor Emerita of Epidemiology, Rollins School of Public Health, Emory University, Atlanta, USA; 5 Département Kimbanguiste de Santé, Société Congolaise de la Pratique Sage-femme, Matadi, République Démocratique du Congo; 6 Société Congolaise de la Pratique Sage-femme, Institut Supérieur des Sciences Infirmières, Kinshasa, République Démocratique du Congo

**Keywords:** Democratic Republic of the Congo, Midwifery, Postabortion care, Abortion induced

## Abstract

**Background:**

Despite a recognized need for midwives to provide post abortion care, there exist barriers preventing them from integrating lifesaving skills such as manual vacuum aspiration (MVA) into practice. This collaborative research with the Professional Association of Congolese Midwives (SCOSAF), sought to understand how certain midwives in the Democratic Republic of Congo (DRC) have overcome barriers to successfully integrate MVA for post abortion care. Specifically, in order to provide locally-driven solutions to the problem of inadequate post abortion care in the DRC, this study aimed to identify examples of positive deviance, or midwives who had successfully integrated MVA in complex working environments following an in-service training facilitated by their midwifery association, SCOSAF.

**Methods:**

Creswell’s mixed method comparative case study design was used to identify positive deviant midwives who had practiced MVA one or more times post training and to explore their strategies and enabling factors. Other midwives who had not practiced MVA post training permitted for a comparison gro cup and further interpretations. Sources of data included a sequential survey and semi-structured interviews.

**Results:**

All 102 midwives invited to be surveyed were recruited and 34% reported practicing MVA post training (positive deviant midwives). No statistical significance was found between the two groups’ demographics and practice facility type. Overall, both groups had positive attitudes regarding midwifery-led MVA and legalization of abortion. Positive deviant midwives demonstrated and described more confidence and competence to practice and teach MVA. They were more likely to identify as teachers and overcome interprofessional barriers by teaching MVA to physicians, medical students and other midwives and position themselves as experts during post abortion emergencies.

**Conclusion:**

Results provided important insight to midwives’ integration of post abortion care in Kinshasa. Strategies used by positive deviant midwives in emergencies allowed them to navigate challenging contexts in order to practice MVA, while simultaneously increasing the credibility of their profession and the dissemination of evidenced-based MVA practice. Programs designed to work with and promote positive deviant midwives as knowledge brokers could be tested for their overall impact on the diffusion of midwifery-led MVA to improve access to safe, respectful reproductive care.

**Supplementary Information:**

The online version contains supplementary material available at 10.1186/s12913-020-05997-7.

## Background

A critical need in many countries is adequate care arising from unsafe abortion practices. Restrictive abortion laws, conflict, displacement, lack of economic autonomy, war-based rape and violence, and legal and cultural restrictions contribute to women’s high rates of unsafe abortions and related complications [1–4]. The maternal mortality rate in the Democratic Republic of Congo (DRC) is one of the highest in the world (846:100000) [5]; deaths due to illicit abortions contribute to at least 7% of all deaths [5, 6]. In 2016, 40,090 women and girls obtained post abortion care with 77% requiring treatment for complications in the capital city of Kinshasa alone [1, 7]. Mortality rates for post abortion complications are as high as 37.8% (95% CI: 23.8–53.5%) [8, 9] owing to critical gaps in community hospitals’ provision of basic delivery services and few referral services for post abortion care [6, 7, 10, 11].

Deaths due to unsafe abortion could be reduced from 22,000 to 9000 [7] per year globally by meeting the need for post abortion care. Certain countries have endorsed the International Confederation of Midwives (ICM) policy on abortion provision [12, 13] by incorporating midwives into abortion-based strategies to reduce maternal mortality [14–19]. This innovative solution includes training midwives in the inexpensive and lifesaving procedure of manual vacuum aspiration (MVA). Countries such as the DRC have high maternal and neonatal mortalities, yet have an available, but under-utilized midwifery workforce [20–22]. Given the context and burden of illicit abortion in the DRC, with thousands of midwives available to be trained, there is great potential for a return on investment if midwives are properly supported in integrating MVA into their practice.

Currently, governmental policies and programs in the DRC have gaps regarding midwifery provision of post abortion care, but there are emerging efforts to address these issues [10, 20]. The national professional midwifery association, SCOSAF (Société Congolaise de la Pratique Sage-Femme) is committed to strengthening its capacity to support members’ integration of manual vacuum aspiration (MVA) into practice. In 2017, an emergency obstetric and neonatal skills (EmONC) continuing education program was created in a joint effort with the Ministry of Health resulting in over 350 midwives trained by SCOSAF in MVA for post abortion emergencies [23]. Post-training evaluations revealed limited integration of MVA overall [24]. However, anecdotally a small number of these trained midwives had found solutions to practicing MVA within challenging environments. To better understand how midwives had been successful, SCOSAF suggested approaching the research question of MVA integration from a solution-based lens. Positive deviance was thus selected as a methodology because its design was suitable for exploring how a small number of midwives had overcome barriers in order to practice MVA.

Contrary to a barrier-focused approach to health services research, positive deviance involves the identification of best practices within a community, resulting in better outcomes [25]. In other words, this approach examines how and why certain individuals have overcome barriers in order to provide local solutions to complex problems [26]. For example, in addressing the high rates of neonatal mortality, one rural community in Pakistan discovered that some midwives whose patients had low rates of neonatal death were naturally substituting old traditional birth practices by placing newborns skin-to-skin following birth [26]. Following this discovery, those positive deviant midwives disseminated their knowledge to other midwives who then adopted the same practice [26]. Other examples include positive deviant nurses in Indonesia who displayed effective communication in family planning, and Rwandan adolescents who successfully negotiated condom use for HIV protection [26–30].

While positive deviancy has been used in similar contexts to explain better maternal health outcomes [31], this approach has not been used to study the integration of post abortion care practice by midwives.^1^ To date, scholarly work on this topic has been largely focused on a deficiencies approach to understand the organizational, technical and professional barriers that prevent MVA integration [17, 32–34]. There are therefore methodological gaps that address how midwives are strategizing to overcome barriers to practice. This research aims to address these gaps in the context of midwifery in order to provide locally-driven solutions to the problem of inadequate post abortion care services in the DRC.

Our approach was to identify and examine “positive deviants” that is, those midwives who overcame barriers to providing post-abortion MVA. Particularly, we aimed to explore those strategies being successfully implemented and the role they had in fostering improvement, and to understand how they compare to midwives who had not yet integrated the skill. We developed the following research questions:

- Who are the positive deviant midwives who integrated MVA into practice post training?
- What strategies and factors facilitated adopting this skill?
- How do they compare to midwives who have not integrated MVA?

## Methods

### Study design

**Fig. 1:**
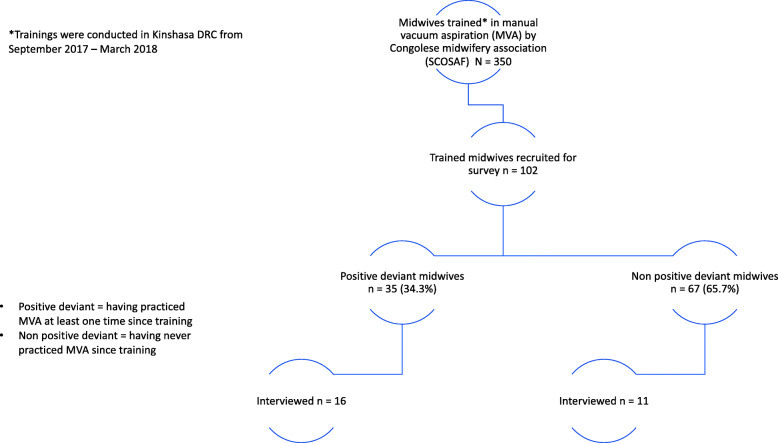
Positive deviant research approach

Positive deviant scholars recommend mixed-method design to identify and describe positive deviant individuals (10). Comparison to non-positive deviant individuals can aid in properly identifying behaviours and factors unique to positive deviants (44). Our research used Creswell’s mixed method comparative case study design to address the research aims [25]. We identified positive deviant midwives who had initiated post abortion care (cases) and midwives who had not yet initiated post abortion care (comparatives) via a quantitative survey of midwives who had received training from their midwifery association (SCOSAF). We analyzed characteristics of each group for convergent or divergent factors [35]. Subsequently, we used a qualitative inquiry via semi-structured interviews with selected members in each case group to explore contextual factors and describe positive deviant behaviours used to achieve MVA integration. Data integration across methods then garnered an understanding of the characteristics of positive deviant individuals and their strategies [35]. Figure 1 shows the comparative case study approach used to identify positive deviant midwives and explore their strategies for MVA practice.

### Ethics approval

Prior to data collection, we obtained ethics approval from the National Committee of Ethics of the Kinshasa School of Public Health in Kinshasa (#ESP/CE/088/2019), Democratic Republic of Congo and the Research Ethics Board (#6017245) at Laurentian University, Canada. Written informed consent was obtained from each participant.

### Population sampled

Midwives considered eligible for the study had completed a 7-day emergency obstetric and neonatal care (EmONC) training facilitated by SCOSAF between September 2017 to March 2018, during which 350 were trained from the nine major health zones in the province of Kinshasa. Trainees worked primarily in labour and delivery and varied in their work contexts and experience.

### Sample size

Sample size of 5% prevalence of post-training MVA practice was based on previous post-training evaluations of Congolese midwives’ report of frequent barriers to MVA integration [24], Previous studies provided little guidance for prevalence due to incomparable midwifery contexts [36]. The minimum sample size for a prevalence of 5% with 95% confidence was 78 midwives. In the event that prevalence of MVA was lower than hypothesized we aimed for one-hundred midwives to be recruited for the survey.

### Case definition, sampling and recruitment

The number of positive deviant midwives (cases) was hypothesized to be few. Therefore, the case group was bound by the deviant behaviour or outcome of practicing MVA for post abortion care one time or more since SCOSAF’s training. The comparison group of non-positive deviants was defined as midwives trained who had not practiced MVA for post abortion care since the training.

The design of our study required a multi-stage sampling approach for recruitment. We sequentially collected two sources of evidence between June 2019 and January 2020 to conduct the case group comparison. In stage one, to maximize the number of positive deviants, the local research team and KMB used the SCOSAF training database to create a list of all trained midwives working at health facilities offering MVA for post abortion care. The team took health facility characteristics into consideration to ensure diverse representation of working environments.^2^ All who were contacted by SCOSAF staff using a pre-approved script agreed to be surveyed. Recruitment ended when the desired sample size was met. Midwives no longer working at the time of recruitment were excluded. Participants were given a self-administered questionnaire that included questions about their post-training practice. Participants were divided into the two groups based on their answer to the question: “Have you ever provided MVA since the EmONC training?”. In stage two, the local research team conducted semi-structured interviews of selected respondents from each case group. We recruited positive deviants to represent the range of MVA integration practices and comparators who best matched the demographics of the positive deviants (gender, age, years of practice).

We combined two context specific tools to create the survey for stage one of the research (Additional file 1). We first took questions from an EmONC training evaluation tool developed by SCOSAF and Ministry of Health in the DRC: “Expérience et confiance en compétences SONU” [38]. This tool had been used by the research team and participants in prior EmONC post training evaluations and included familiar and comprehensible terminology. Next, we included the IPAS “values clarification and attitudes transformation” survey, with permission, to measure the attitudes of midwives about providing post abortion care [39, 40]. Together, this created a tool that provided data representing the range of factors proven to influence provider provision of post abortion care: socio-demographic characteristics, facility characteristics, provider training and experience, professional confidence, and personal attitudes and values towards abortion. The self-administered survey was in French (a national language) and was administered in a neutral setting, with local researchers available to provide additional explanations in the more common national language of Lingala. For stage two, the semi-structured interview schedule explored barriers and facilitators to practice and how midwives were able to practice MVA (Additional file 2). Our local research team audio recorded the interviews and conducted them in a neutral setting in Lingala or French.

### Analytic strategy

Both the positive deviant methodology and Rogers’ Theory of Diffusions of Innovations Theory informed our analysis [26, 41]. The rationale for a positive deviant approach to MVA integration is that it aims to explain the actions of the few who, when faced with a problem or adversity, create solutions. Their solutions then can be organized and made accessible to the majority so they can have the tools needed to succeed [26, 42]. Success in confronting adversity depends in part on a combination of individual-, political-, health- and social-systems that positively or negatively impact an individual’s or organization’s ability to implement change [43]. Roger’s Theory of Innovations provides a framework from which to analyze these complex factors and explain how positive deviant midwives have succeeded. Positive deviant applications of Rogers’ theory represent a collaborative, community approach that can leverage local and feasible methods for disseminating innovations [44].

**Fig. 2:**
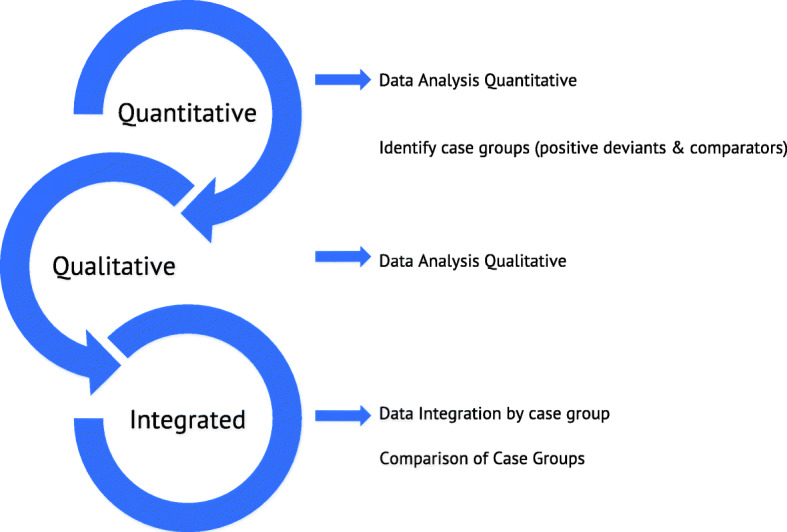
Mixed methods case comparative research design

We used Rogers’ three basic clusters of concepts as framework for the analysis and integration of data: 1) perceptions of the innovation; 2) characteristics of individuals who do or do not adopt the innovation; and 3) the context in which the innovation is introduced [45]. Within this framework, specific themes emerged from the data, reflecting both top down and bottom up approaches [25]. The intent of our study design was to integrate across methods and between cases in order to understand the characteristics of positive deviant individuals and identify their strategies [35]. Data analysis for each source of evidence occurred separately prior to the case group analysis and then were compared in the final interpretation (Fig. 2).

We descriptively summarized the survey responses from stage one for the overall sample of respondents. Participants’ characteristics included socio demographics, professional experience, MVA practice, knowledge and attitudes regarding their role expansion and willingness to provide MVA for post abortion care. Questions regarding attitudes and values of MVA were measured using a Likert scale, which were added to create an overall score [39, 46]. Participants’ MVA experience included observing or assisting in MVA procedures, perceived competence and confidence to practice and teach MVA, and perceived barriers and facilitators to practice. Their facility’s characteristics included EmONC services provided, hospital governing authority, and facility supplies.

Using SPSS software, we conducted a bivariate analysis between case and comparator groups. We used chi-square tests for categorical variables. Due to non-normal distribution of attitudes and values about MVA, we analyzed for differences in the groups’ scores using the non-parametric Mann Whitney-U test. Probabilities of less than 0.05 rejected the null hypothesis of no differences between the two groups.

Semi-structured interviews conducted in Lingala were forward translated to French and back translated by another researcher to ensure conceptual equivalence (AL, AM) [47]. Interviews conducted in French were transcribed directly and verified by the principal researcher (KMB). The interviews were uploaded in NVivo and a code structure was created using the three basic clusters of influence as outlined by Rogers’s theory [45]. The first five transcripts were coded independently by KMB, AL and AM, and results were compared to ensure accuracy and to agree upon any new codes that emerged from the data. All discrepancies in the analysis were resolved together as a research team.

Data integration occurred at three points: The first point involved analysis of: 1) survey results between the groups of positive deviants and comparators; 2) qualitative findings from interviews between the two groups; and 3) a side-by-side comparison of results from both sources of evidence to locate convergences or divergences while exploring factors associated with positive deviant behavior. Second, integration occurred when results for each theme were compared between the groups. The third integration involved producing a narrative and joint display.

### Research collaboration regarding abortion

In the DRC, abortion is legally restricted. As a result, clandestine abortions are common and stigmatized [7, 8]. Our team gave strict attention to maintaining local collaboration, which is crucial in building trusting relationships and facilitating trustworthiness, particularly when researching topics that intersect with abortion [48]. This was achieved by creating a stakeholder committee comprised of governmental and non-governmental experts in midwifery and abortion care and with a team of two Congolese midwife researchers.^3^

As midwives belonging to the midwifery association, the team of researchers held a unique and important insider perspective regarding the provision of post abortion care, and a high degree of cultural sensitivity and understanding in relation to the research topic (37). Cultural competence is known to increase the researcher’s ability to properly represent reality and foster trust with participants in abortion research contexts [49]. Further, culturally competent researchers can self-reflect and recognize their own biases [49]. We therefore considered this insider perspective as contributory to the trustworthiness of the research. An outside researcher might cause discomfort to the participants and create an environment where they felt intimidated and judged.

## Results

### Characteristics of participants

1. A total of one hundred and two (*n* = 102) midwives participated in the study. The social, demographical and professional characteristics of the participants and subset are presented in Table 1. Thirty-five (35) of the 102 participants had practiced MVA since being trained (34.3%). Of those midwives 21 (20.6%) applied it for the first time, and 14 (13.7%) had improved their technique since the training. Prevalence is presented in Table 2 and all bivariate associations of the two case groups are presented in Table 3.

**Table 1:** Characteristics of participants*

	Survey Respondents (*n* = 102)	Interviewed (*n* = 27)
**Demographics of Participants**	n (%**)	**n (%)**
Gender		
Male	9 (8.8)	4 (14.8)
Female	93 (92.2)	23 (85.2)
Age		
20–39 years	40 (40.8)	9 (33.3)
40–59 years	58 (59.2)	18 (66.6)
Years as midwife		
15 years and less	24 (24.2)	16 (59.3)
More than 15 years	75 (75.8)	11 (40.7)
Type of Midwife		
Midwife	65 (63.7)	18 (66.7)
Auxiliary midwife	37 (36.2)	9 (33.3)
Teaching (official or otherwise)	27 (26.5)	8 (29.6)
Other MVA training	25 (25.3)	9 (33.3)
Other EmONC training	29 (28.7)	8 (29.6)
**Hospital Characteristics**		
Type of EmONC services		
Comprehensive	86 (85.1)	24 (88.9)
Basic	15 (14.9)	3 (11.1)
Operating Authority		
Government	86 (84.3)	17 (63)
Private	9 (8.8)	5 (18.5)
Catholic (Mission)	7 (6.9)	5 (18.5)
Offers MVA for post abortion care	91 (90.1)	24 (88.9)
Consistent supplies MVA	65 (63.7)	21 (77.8)

*The sample was chosen from a purposive list of 350 midwives trained by SCOSAF in MVA in either 2017 or 2018, working in facilities offering MVA for post abortion care. All midwives contacted to participate in the study agreed to be surveyed

**Percentages among known responses

**Table 2:** Variations of MVA practice

	Survey Respondents (*n* = 102)	Interviewed (*n* = 27)
**Prevalence of MVA**	**n (%)**	**n (%)**
1st time since MVA training	21(20.6)	7 (25.9)
Better since MVA training	14 (13.7)	9 (33.3)
Never	67 (65.7)	11 (40.7)
**Frequency of MVA practice**		
One time ever	17 (16.7)	8 (29.6)
Every 6 months	6 (5.9)	2 (7.4)
Every month	12 (11.8)	6 (22.2)
n/a	67 (65.7)	11(40.7)

**Table 3:** Bivariate associations between positive deviants and comparators

	Positive Deviant Midwives (*n* = 35)	Non-Positive Deviant Midwives (*n* = 67)	
	n (%)	n (%)	***p*** value
**Demographics**			
Age			
20–39 years	11 (33.3)	20 (30.8)	0.80
40–59 years	22 (66.7)	45(69.2)	
Years as midwife			
15 years and less	21 (61.8)	42 (64.6)	0.78
More than 15 years	13 (38.2)	23 (35.4)	
Type of Midwife			
Midwife	23 (65.7)	42 (62.7)	0.76
Auxiliary midwife	12 (34.2)	25 (37.3)	
**Professional Experience**			
Teaching (official or otherwise)	16 (45.7)	11(16.7)	0.002*
Year trained in EmONC			
2017	17 (48.6)	23 (36.5)	0.24
2018	18 (51.4)	40 (63.5)	
Teaching (official or otherwiseOther MVA training	15 (44.1)	10 (15.4)	0.002*
Other EmONC training	13 (37.1)	16 (24.2)	0.17
Additional MVA experience			
Observed 1st since training	11 (31.4)	21 (31.3)	0.99
Observed prior training	6 (17.1)	12 (17.9)	0.92
Assisted 1st since training	9 (25.7)	17 (25.4)	0.97
Assisted prior training	14 (40.0)	11 (16.4)	0.009*
MVA for therapeutic abortion			
Yes	12 (34.3)	3 (4.6)	< 0.0005**
Not comfortable answering	7 (20.0)	17 (26.2)	
**Confidence**			
Level of confidence to practice MVA			
Very confident to practice	24 (68.6)	10 (17.9)	< 0.0005*
Not very confident	11 (31.4)	46 (82.1)	
Perceived competence to practice MVA	31 (91.2)	39 (59.1)	0.001*
**Barriers and facilitators**			
Barriers			
Lack of interprofessional support	9 (25.7)	11 (16.4)	0.26
Lack of MVA supplies	11 (31.4)	38 (56.7)	0.02*
Lack of MVA aspirator	11 (31.4)	32 (47.8)	0.11
Facilitators			
SCOSAF	32 (91.4)	46 (68.7)	0.01*
Ancillary support to practice			
Mentorship	14 (40)	32 (47.8)	0.46
MVA equipment	17 (48.6)	41 (61.2)	0.22
**Facility characteristics**			
EmONC services			
Comprehensive	29 (82.9)	57 (86.4)	0.64
Basic	6 (17.1)	9 (13.6)	
Operating Authority			
Government	27 (77.1)	59 (88.1)	0.15
Private/Mission	8 (22.9)	8 (11.9)	
Consistent supplies for MVA	27 (77.1)	38 (56.7)	0.04*

*factors not included if cell size was smaller than 5 units

*significant if *p* < 0.05

### Case study results

A subset of twenty-seven (27) participants were interviewed. We reached saturation with 16 positive deviants and 11 comparators. No potential positive deviants refused to be interviewed, but 11 comparators selected for interviews could not be reached due to changes in contact information. All comparators reached agreed to participate. Table 4 presents a joint display of qualitative and quantitative results across groups, with data organized into the three themes derived from observations based on the original framework:

**Table 4:**
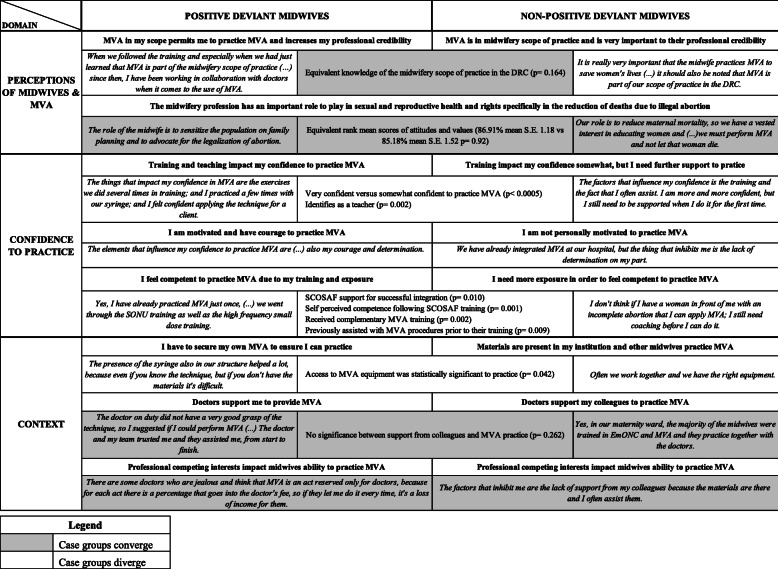
Thematic case groups comparisons (see legend)

2.1) Midwifery perceptions of MVA, abortion and midwifery scope of practice, 2.2) Midwives’ confidence and competence of MVA, and, 2.3) Immediate working context. Each category was further divided into sub-themes. The following narrative is an interpretation of data integration and a comparison of positive and non-positive deviant midwives by categoric theme. Divergences and convergences are additionally explored.

### Perceptions of MVA

#### Midwifery scope and MVA

In the survey, groups demonstrated equivalent knowledge of the midwifery scope of practice in the DRC (*p* = 0.164). The interviews revealed similar changes across groups in perspective and knowledge of midwives’ role with regards to post abortion care from SCOSAF’s MVA training. Specifically, they expressed the opinion that midwives’ involvement in MVA practice increased the credibility of their profession and is an essential skill, as midwives are well positioned and capable of saving the lives of women who had undergone clandestine abortions.

In relation to their ability to integrate MVA, positive deviants described how SCOSAF’s training allowed them to fully understand their autonomy and the key role they hold within the health care system to decrease maternal mortality due to unsafe abortions. This newfound empowerment and self-perception as an MVA provider were catalysts to practice and evolved their own professional identity in favour of practicing MVA:

*Previously, this practice in our structure was reserved only for doctors (...). When we followed the training and especially when we had just learned that MVA is part of the field of midwifery practice, (..) since then I work in collaboration with the doctors when it comes to the use of MVA*^4^*(case group 1: participant 1).*

Positive deviants also associated their practice of MVA with its overall positive impact on midwifery by increasing midwives’ credibility with colleagues and clients. They saw MVA practice as honourable and important for midwifery and the advancement of maternal and child health. Indeed, this new sense of autonomy and credibility allowed them to advance their own position as midwives within their hospitals, often evolving into leadership roles and propagating the use of MVA:

*It gave me value and consideration in the eyes of others...I am happy to be the champion and leader in my structure, because it is thanks to me that everyone practices MVA (case group 1: participant #14).*

Comparators had similar knowledge and views of MVA within their scope of practice. In interviews they revealed no negative opinions or views regarding MVA being within the scope of midwifery practice for post abortion care. However, they did not apply this to their own personal practice or lack thereof:

*It is really very important that the midwife performs MVA to save the lives of women who die every day as a result of abortions...it should also be noted that MVA is part of the scope of midwifery in the DRC (Case group 2: Participant 25).*

#### Attitudes about post abortion care

Overall, midwives expressed positive attitudes and professional intent to practice post abortion care. In the survey, midwives scored similarly to questions regarding their willingness to provide post abortion care, to be publicly known as a post abortion provider, and their beliefs of abortion legislation. Mean scores were high at 86.91% (mean S.E. 1.18) for positive deviants and 85.18% (mean S.E. 1.52) for comparators, with no statistical difference between the rank mean scores (*p* = 0.92). During interviews, both groups acknowledged the rate of unwanted pregnancies as too high in their country, contributing to higher levels of deaths due to clandestine abortions. They expressed belief that midwives must address this problem by advocating for better access to contraception, decreasing illegal activities and improving abortion laws. Both groups spoke of inequities in their health care systems that lead to unsafe abortions, that the system was unfair and had a negative impact on women and girls.

*I think that many girls or young people resort to clandestine abortions because the Ministry of Health has not set up a safe abortion process, many women do these abortions because their pregnancies are unwanted (case group 1: participant 1).*

Midwives discussed the importance of improving their country’s policies and programs to improve access to birth control and abortion and were clear that midwifery needs to be present at all levels of implementation:

*Midwives have a very crucial role to play in raising women’s awareness on family planning but also, we have to advocate for the correct application of the Maputo Protocol, and also participate in decision making regarding women’s health. (case group 2: participant 25).*

### Midwives’ confidence and competence of MVA

#### Confidence

In the survey, midwives were asked to rank their confidence to practice and teach MVA post training. Positive deviants were more likely than comparators to report being very confident, while comparators were more likely to be somewhat confident to practice MVA (*p* <  0.0005). Positive deviants were more likely than comparators to hold a formal or informal teaching role (*p* = 0.002). They were also more likely to express confidence in their ability to teach MVA (64.2% compared with 94.3% of comparators expressing only somewhat confidence to teach MVA).

In the interviews, midwives were asked to describe their levels of confidence including what factors impacted their confidence to practice MVA. Positive deviants used words such as self-worth, personal conviction, motivation and courage to describe the internal or inherent factors that increased their confidence to practice, particularly with regards to the first time they practiced MVA.

*My experience for the integration of MVA is only the fruit of my conviction and my courage, a case arrived and the emergency doctor didn’t know what to do and he left me in charge (case group 1: participant 2).*

Midwives described that successfully performing MVA for the first time under extremely stressful conditions reinforced their confidence by positively impacting their self-respect, self-worth and perceived place within their interdisciplinary team. This new confidence as a team member led them to share their knowledge by teaching and training others to use MVA. Being a teaching resource or reference point in their hospital also positively impacted their confidence as practitioners.

*My level of confidence is stable because I master the technique perfectly and I also train others (...) I have even become a leader in my structure (case group1: participant 6).*

Conversely, comparators described an inherent lack of motivation or interest to practice MVA or to ask someone to mentor them. Midwives suggested that it was purely a matter of self-determination and a personal decision to motivate themselves to practice or to ask for help:

*I think I’ve mastered the technique with what I see from my colleagues. But I just lack determination and a little bit of decisiveness because I wasn’t interested in it at all (case group 2: participant 19).*

Some described that their confidence to practice was poor due to the length of time since their training, or lack of exposure in clinical practice. This led to needing more mentorship or supervision, or even more training so they could be confident enough to practice:

*The element that impact my confidence is the lack of practice after the training because if you have learned something and you don’t practice, you forget certain steps, but I would need someone to assist me the day I do the MVA (case group 2: participant 22).*

Many comparators were working in contexts where MVA was practiced by either physicians or other midwives. They seemed to be content to support their colleagues in MVA procedures instead of performing MVA themselves. However, if faced alone with a clinical situation that demanded MVA, they often described full intent to provide the necessary care, regardless of their confidence levels:

*Yes, I would like to practice MVA 1 day and I know that 1 day I will do it because with the EmONC training and as I already assist those who do it if a case happens and I am alone I will do it. (case group 2: participant 18).*

#### Confidence and competence

The most common external factor that influenced midwives’ confidence to practice MVA was their SCOSAF training. In the survey, positive deviants were more likely than comparators to report they felt competent following the training (*p* = 0.001). They were also more likely to identify SCOSAF as the primary source of support for successful integration (*p* = 0.010). In the interviews, both groups discussed the importance of receiving MVA training from SCOSAF and its positive impact on their competence. Discussions differed between the groups based on how much the training and perceived competency impacted their confidence to actually practice on their own. The comparators expressed more reticence towards practicing and discussed needing ancillary supports before they felt completely confident:

*The factor that impacts my confidence is the EmONC training (...) but I still need to be supported by the experts when I do it for the first time (box group2: participant 20).*

However, perceived competence achieved from the training to practice MVA did impact positive deviants’ practice, compelling them to apply their knowledge:

*The elements that impact my confidence in MVA are the exercises that we did several times at the training; and when I came home, I practiced a few times with our syringe; and I felt confident applying the technique to a client (case group 1: participant 1).*

In the survey, length of time since being trained (2017 or 2018) was not associated with whether midwives had initiated MVA practice (*p* = 0.24), however interviews with comparators revealed that their confidence had waned over time:

*At the moment my confidence level is a bit low (...) which means that for the moment before practicing I first have to redo the theoretical and practical training we received at SCOSAF (case group 2: participant 23).*

Another influence appeared to be the amount of training and experience midwives had had prior to the training. The survey revealed that positive deviants were more likely than comparators to have previously assisted with MVA procedures prior to their training (*p* = 0.009) and to have received complementary MVA training (*p* ≤ 0.002).

### Context

Results for context are divided into two categories addressing: 1) the midwives’ immediate work environments such as supply and equipment and 2) working relationships, particularly in the working environment at the actual moment of the emergency or MVA practice.

#### Access to MVA equipment

MVA integration did not differ whether midwives were working for public or private entities nor if their hospital provided basic or comprehensive EmONC care. During the interviews, several participants referred to their hospital-specific training which had left mannequins for training and equipment at their disposal.

Having immediate access to MVA equipment was statistically associated with practice (*p* = 0.042). MVA equipment was either made available by the hospital, which in many cases was due to the donations of an organization, belonging to a medical colleague, or the midwife themselves. Midwives described MVA availability as inconsistent, caused by stock outages or doctors taking the equipment home with them. This midwife described how she stowed her own MVA to guarantee consistent access in emergencies:

*Then we brought her to my office and as all the materials are in there, I suctioned out the contents and in a few minutes the woman was saved (case group 1: participant 6).*

#### Relationships and access to MVA

While the survey found no significance between support from colleagues and MVA practice (*p* = 0.26), both groups discussed the importance of care provider relationships throughout the interviews. Predominantly, midwives discussed who was present or not during emergencies making clear that the interprofessional encounter was more relevant than overall institutional environment. Interprofessional relationships were most often with physicians followed by medical students or midwives.

Positive deviants in the private sector revealed competing financial interests between themselves and physicians. More complicated procedures, such as dilatation and curettage, cost more for a patient and therefore, were more profitable for physicians. When physicians were present, midwives were less likely to manage the client’s care. MVA profits also caused interprofessional tensions, resulting in a reluctance on the part of some physicians to allow midwives to perform post abortion care.

*There are some doctors who are jealous and think that MVA is a procedure reserved only for doctors, because for each procedure there is a percentage that goes into the doctor’s fee; so, if they let me do it every time, it’s a loss of income for them (case group 1: participant 1).*

For comparators, competing interests between physicians and midwives and their own motivation to practice were described simultaneously. While they expressed a desire for more support from their peers, they often stated that midwives and physicians were already practicing in their setting. They described both a need for their peers to be more inclusive of them, while admitting they could also be more confident and motivated to include themselves:

*The factors that inhibit me are the lack of support from my colleagues because the materials are there and I often assist them, but I think that I too must have the courage (case group 2: participant 18).*

Many comparators discussed collaborations with their peers in order to assist them during emergencies and described a sense of accomplishment in these collaborations in order to save a life:

*the woman was bleeding so badly that we couldn’t wait many minutes because her life was in danger. I and the other midwife had decided to do the MVA (...), and we aspirated (...) I actively assisted and we were able to save that woman’s life (case group 2: participant 26).*

Positive deviants also described their interactions with physicians during emergencies as educational encounters. If the physician did not know the technique, the midwife would capitalize on the occasion to inform and teach. Midwives discussed how they were in many cases able to convince their peers of the benefits of MVA during the emergency:

*The doctor who was there asked that we could prepare the material for the curettage, as I had just been trained and we still had the MVA syringe that had never been used; I proposed to the doctor that we could first try the new aspiration technique; after my explanations on the advantages of the technique, they accepted and I did so in front of the doctor and my colleagues who also learned that day (case group 1: participant 14).*

*The doctor on duty did not have much knowledge of the technique, so I suggested if I could perform MVA. The doctor and my team trusted me and assisted me from start to finish (case group 1: participant 15).*

Teaching during one emergency could lead to the midwife teaching others as other emergencies arose:

*I did it 1 day in an emergency and the doctor was busy, and we saved this woman together with the trainee doctor...I master the technique well and I can do it alone without supervision and I teach the other doctors to do it (case group 1: participant 5).*

## Discussion

Our research found that positive deviants encompassed all ages, types of midwives, experience levels and facilities. While their perceptions of the scope of midwifery and MVA for post abortion care differed little from comparators, one distinction is clear. In an emergency, positive deviants relied on their self-confidence fueled by motivation, sense of professional credibility and competence. In each story, positive deviants described an emergency that became a critical juncture leading them to initiate MVA. With MVA equipment at hand and the right combination of inter- or intra-professional support, those midwives were able to push past institutional and cultural norms regarding their role in post abortion emergencies. Faced with a clinical emergency, this combination of internal and external factors propelled them to courageously apply the MVA technique.

Positive deviants further leveraged their confidence as MVA practitioners and as teachers in order to inspire their colleagues to trust in their expertise and to learn the MVA technique from them. Examples included convincing colleagues to abandon dilatation and curettage, a riskier yet commonly practiced skill, and teaching the MVA technique to physicians and residents. This strategy of positioning themselves as mentors in an emergency allowed them to momentarily transcend the traditional patriarchal structures of their institution and health system. This non-threatening approach was acceptable to their physician colleagues in the moment. Following this event, midwives evolved into a mentor and in some cases an expert in their institution.

Our findings are similar to a study of integrating MVA for post abortion care in Ghana. Post training, investigators reported a similar prevalence (31% *p* = 0.015) [36]. In that study, working in private facilities was the only association across midwife characteristics and MVA practice. The prevalence of midwifery MVA integration was lower than among physician participants (80%), which authors attributed to organizational and professional barriers. Mistrust of midwives and lack of clear definition of the midwifery model of care also contribute to the midwife’s difficulty practicing the new skill [33, 50].

Our qualitative results mirror a systematic review regarding task shifting in midwifery, where sharing a skill across traditional professional boundaries challenged hierarchical structures [33, 50]. Abortion is typically a medical act. Dilatation and curettage was historically the preferred method for post abortion care, and is a highly medicalized event [51]. MVA is a newer, simpler, safer and less technical skill [51] that can be conducted by midwives, in all facilities that offer birth [51]. As the skill is proven to be less technical, it therefore becomes unjustifiable to withhold its use from midwives, yet there remains a real medical resistance and mistrust of midwives’ capacity to manage abortion care [19, 52]. In a survey of midwives at an international midwifery congress 41% of midwives qualified to provide MVA reported that they were prevented from practicing, listing medical professional and organizational structures as barriers [32].

While our research found that physician support and trust were conducive to midwifery practice across both positive deviant and comparator groups, our research adds an important insight to explain how midwives navigated challenging professional boundaries and in doing so, increased the trust and corroboration of their medical peers. Similar to some previous studies, confidence is an important starting point [34, 53]. However, we also found that confidence was enhanced by positive deviants’ motivation and interest in practicing MVA, leveraged by their strong sense of professional identify and scope of practice and their perceived competence and knowledge of the skill. Comparators were less confident and motivated to practice, while still remaining supportive.

**Fig. 3:**
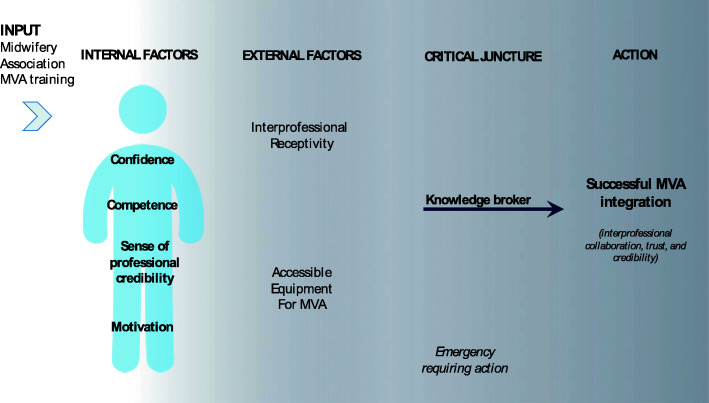
Proposed framework for positive deviant midwives’ integration and diffusion of MVA

Our research provides further insight into how midwives trained in MVA challenged and overcame interprofessional barriers by strategically positioning themselves in a clinically urgent situation, making a calculated risk to save a life. Positive deviants presented themselves as intermediaries or knowledge brokers, promoting the practice of MVA to their peers, a still uncommon practice in their context, while subtly demonstrating the role of the midwife in a post abortion emergency. Figure 3 represents a proposed framework explaining how positive deviants challenged professional boundaries, integrated MVA and increased the trust of their medical peers. Knowledge brokers in similar contexts to the DRC have been shown to successfully disseminate information based on their ability to actively seek opportunities to demonstrate evidence-based practice, network and build relationships, and adapt to the immediate context [54]. Like knowledge brokers, positive deviants were driven by their internal motivation and beliefs of midwifery and sexual and reproductive rights. They had the confidence to creatively adapt in an emergency and to act quickly in a non-threatening manner, overcoming professional and gendered norms while simultaneously raising awareness and credibility of the midwifery profession.

### Strengths and limitations

Three main strengths of this study include: 1) the mixed method case study design that allowed for an in-depth analysis of the “who” and “how” Congolese midwives integrate MVA into practice; 2) the positive deviant approach that allowed for identifying pragmatic solutions that overcome well documented professional barriers to MVA integration; and 3) the collaborative approach that provided SCOSAF with vital information to leverage midwifery in the context of abortion care in their country.

The main limitation of the study was due to the coronavirus pandemic. Travel and ethical restrictions meant a required adaptation to the final step of study. Rather than employing a focus group discussion for validation, results were electronically shared with stakeholders, with dissemination plans occurring at a later date. Another limitation occurred during the data collection period. Frequent deployment of researchers (AL, AN) to teach in rural areas caused delays in recruitment for the semi-structured interviews for comparator midwives. During this time lapse, changes in participants’ contact numbers resulted in some comparators being no longer reachable. Contactable comparators were easily recruited for the qualitative phase.

## Conclusion

This is the first study to independently examine midwifery integration of MVA in the DRC after training provided by the midwifery association. There is an important link between SCOSAF, the results of our study and the development of the midwifery workforce as it pertains to abortion. Midwifery associations act as a focal point for the profession, ensuring quality midwifery themed training and providing educational support. SCOSAF’s training permitted midwives to understand their professional scope and to develop an openness to post abortion care. Future research is needed to explore the link between the impact of midwifery-led training and supports, professional identity and MVA integration. Further, SCOSAF could collaborate with positive deviants to continue to diffuse midwifery-led MVA and grow their capacity in-house to support midwives in abortion care. As knowledge brokers and leaders, positive deviants could properly support midwives who are interested in providing post abortion care, yet who are less confident or competent to practice. Midwifery associations also act as a voice to represent the profession at local and national levels [55]. Midwives’ associations must be involved in the policymaking process related to sexual and reproductive health and rights. Yet the extent of their contribution is hindered by a lack of professional recognition or understanding of the roles of midwives in health and political systems. Study results have important implications for SCOSAF as much as it does their membership, allowing them to leverage technical expertise to advocate for relevant and meaningful contributions to governmental and bilateral investments in abortion in the DRC.

## Supplementary Information

**Additional file 1.**

**Additional file 2.**

## Data Availability

The datasets generated and/or analysed during the current study are not publicly available because it belongs strictly to the Association of Congolese Midwives (SCOSAF), but are available from the corresponding author on reasonable request.

## Abbreviations

MVA: Manual vacuum aspiration
DRC: Democratic Republic of Congo
ICM: International Confederation of Midwives
EmONC: Emergency obstetric and neonatal skills
SCOSAF: Professional Association of Congolese Midwives

## Footnotes

1 KMB consulted with a positive deviant researcher regarding research design.

2 Hospitals were classified based on WHO signal functions to provide routine and emergency obstetric and neonatal care (EmONC) and their governing authority [37]

3 The stakeholder committee comprised of governmental and non-governmental experts in midwifery and abortion care including SCOSAF, PNSR (programme national de la santé reproductive), SCOGO (Société Congolaise de Gynécologie et d’Obstétrique), UNFPA (United Nations Population Fund) and IPAS (International NGO for reproductive rights and health). This committee provided guidance at various points during the research process, including local ethics approval, research design, data collection instruments, data analysis and results.

4 All citations were translated to English.
