# Supplementary material for: Midwives’ integration of post abortion manual vacuum aspiration in the Democratic Republic of Congo: a mixed methods case study & positive deviance assessment
Source: BMC Health Serv Res. 2020 Dec 10;20:1136. doi: 10.1186/s12913-020-05997-7 (PMC7726277; doi:10.1186/s12913-020-05997-7)
Supplement: Supplementary file 1 — Additional file 1. [file 12913_2020_5997_MOESM1_ESM.docx]

**translated from French*
**Midwives’ Integration of Post Abortion Manual Vacuum Aspiration in the Democratic Republic of Congo: A Mixed Methods Case Study & Positive Deviance Assessment**

_________________________

Please answer the following questions based on your current knowledge and values.

Please do not put your name on this form.

You will create a unique identifier to allow us to match your answers before and after the survey while maintaining your confidentiality.

Responses without identifying information may be used for evaluation purposes and for professional presentations and publications.

|  | # of sisters | Month of birth | 3 last numbers of telephone # | Name of your province of birth |
| --- | --- | --- | --- | --- |
| Example | *2* | *10* | *034* | *Kinshasa* |
| **Your unique identifier** |  |  |  |  |

1. Province of your hospital : 🞎 Kinshasa 🞎 Kongo Central 🞎 other
2. EmONC services : 🞎 Comprehensive 🞎 Basic
3. Operating authority: 🞎 Private 🞎 Public 🞎 Catholic 🞎 Other
4. Rural or urban : 🞎 urban 🞎 rural
5. Type of midwife : 🞎 Midwife (A1) 🞎 Nurse midwife (A2)

🞎 Auxiliary midwife (A3) 🞎 nurse 🞎 other

1. Work in labour and delivery 🞎 yes 🞎 non – if no indicate service : _______________
2. Do you teach ? 🞎 yes 🞎 no
3. How long have you been a midwife?

🞎 Less than 5 years 🞎 6 – 15 years 🞎 more than 15 years

1. Age : 🞎 20-29 years 🞎 30-39 years 🞎 40-49 years 🞎 50-59 years 🞎 60-69 years
2. Man 🞎 Woman 🞎
3. Year you were trained in EmONC (MVA) by SCOSAF:

**2017 ou 2018**

1. Other EmONC training 🞎 yes 🞎 no

If yes, date: ___________ (month/year)

1. Other MVA training 🞎 yes 🞎 no

If yes, date : : ___________ (month/year)

1. Other MVA training before or after SCOSAF training? Yes/no?
2. Since SCOSAF training, have you practiced MVA?

🞎 Yes, I practiced MVA for the first time since the training

🞎 I practiced before, but have practice since the training

🞎 No, I have not practiced MVA

1. Have you observed and/or assisted with an MVA procedure before?

🞎 Yes, I observed for the first time since the training

🞎 Yes, I assisted for the first time since the training

🞎 No, I have not assisted nor observed since the training

🞎 I assisted before the training

🞎 I observed before the training

1. How many times do you practice MVA since the training?

🞎One time 🞎Every six months 🞎Every month 🞎n/a

1. Who supported you to practice MVA (check all that apply) ?

🞎 Supervisor

🞎 Collègues

🞎 Administration

🞎 SCOSAF

🞎 Other

1. What were the barriers that prevented you from practicing MVA (check alll that apply) ?

🞎Workload

🞎Lack of time

🞎Lack of supervision

🞎Lack of support from colleagues

🞎Lack of equipment and supplies

🞎No MVA

🞎 Other

1. Do you feel that your hospital supports the use of MVA? 🞎 yes 🞎 no
2. Do you feel that your hospital offers MVA for post-abortion care? 🞎 oui 🞎 non
3. Do you feel that your hospital has the equipment to offer MVA for post abortion care ? 🞎 oui 🞎 non
4. In my country, midwives are authorized to use MVA ?

True

False

I don’t know

1. In my country, midwives are authorized to provide post abortion care?

True

False

I don’t know

1. What is your confidence level to practice MVA?

🞎Very confident, I don’t need support

🞎Somewhat confident, I need support and mentorship

🞎Not confident, I don’t feel confident to practice this skill

🞎The politics of my hospital do not allow me to practice MVA

1. What is your confidence level to teach MVA?

🞎Very confident, I don’t need support

🞎Somewhat confident, I need support and mentorship

🞎Not confident, I don’t feel confident to teach this skill

🞎The politics of my hospital do not allow me to practice MVA

1. Did the training you received by SCOSAF provide you with the competence you required to practice MVA?

🞎 yes 🞎 no

1. What support do you require to practice MVA?

🞎Further mentorship

🞎Further training

🞎Equipment

🞎Time

🞎Other (specify) ______________

1. Have you provided a therapeutic abortion using MVA since the training by SCOSAF?

🞎 yes 🞎 no 🞎 I prefer to not answer

1. I will provide the principles of respectful care for women seeking post -abortion services 🞎 yes 🞎 no

**Answer the following questions to the best of your ability, if it does not apply leave the questions blank**

|  | Completely Disagree | Disagree | Neither agree of disagree | Agree | Completely Agree |
| --- | --- | --- | --- | --- | --- |
| The issue of abortion is of little importance to me | 1 | 2 | 3 | 4 | 5 |
| I support the provision of family planning and contraceptive  services in my country. | 1 | 2 | 3 | 4 | 5 |
| I feel comfortable working to increase access to family planning  and contraceptive services in my country. | 1 | 2 | 3 | 4 | 5 |
| I support the provision of abortion services as permitted by law  in my country. | 1 | 2 | 3 | 4 | 5 |
| I feel comfortable working to increase access to abortion  services as permitted by law in my country. | 1 | 2 | 3 | 4 | 5 |
| I feel comfortable talking with my family and friends about my  involvement with post abortion care. | 1 | 2 | 3 | 4 | 5 |
| I would feel comfortable observing a post abortion procedure. | 1 | 2 | 3 | 4 | 5 |
| I would feel comfortable performing or assisting a post abortion  procedure. | 1 | 2 | 3 | 4 | 5 |
| I am clear about my personal values concerning abortion. | 1 | 2 | 3 | 4 | 5 |
| I feel very conflicted about abortion. | 1 | 2 | 3 | 4 | 5 |
| I can clearly explain my personal values concerning abortion. | 1 | 2 | 3 | 4 | 5 |
| I can respectfully explain values concerning abortion that  conflict with mine. | 1 | 2 | 3 | 4 | 5 |
| I feel empathy for women who have experienced abortion. | 1 | 2 | 3 | 4 | 5 |
| All women should have access to safe, comprehensive  abortion care in the first trimester. | 1 | 2 | 3 | 4 | 5 |
| Access to first-trimester abortion should be restricted to  certain circumstances. | 1 | 2 | 3 | 4 | 5 |
| All women should have access to safe, comprehensive  abortion care in the second trimester. | 1 | 2 | 3 | 4 | 5 |
| Access to second-trimester abortion should be restricted to  certain circumstances. | 1 | 2 | 3 | 4 | 5 |

1. Any other comments ?
