# Supplementary material for: Midwives’ integration of post abortion manual vacuum aspiration in the Democratic Republic of Congo: a mixed methods case study & positive deviance assessment
Source: BMC Health Serv Res. 2020 Dec 10;20:1136. doi: 10.1186/s12913-020-05997-7 (PMC7726277; doi:10.1186/s12913-020-05997-7)
Supplement: Supplementary file 2 — Additional file 2. [file 12913_2020_5997_MOESM2_ESM.docx]

**Midwives’ Integration of MVA**

**Interview Guide**

**Translation from French*

| Interviewer: ____________________________________  Case Group:  Integrated MVA □  Not integrated MVA □ |
| --- |
| Participant code: ____________________________________  Location of interview: ____________________________________  Date of Interview: ____________________________________  Time of Interview: ____________________________________ |

Remind the interviewee that the interview is being recorded, but that it will be anonymized.

Thank you for collaborating with us on this research.

There are two parts to the interview:

The first covers your experience with the integration of MVA since you were trained.

The second invites you to provide information about yourself.

Thank you.

**Part 1: semi-structured interview**

1. **Have you practiced MVA (manual vacuum aspiration) since the training?**

*Invite the interviewee to elaborate.*

1. **A. If yes, describe one or more of your experiences with MVA integration.**

**B. If no, would you like to practice MVA? Are there midwives that practice MVA at your hospital? Describe what you have observed?**

1. **What do you think are the elements or strategies that make it easier for you to practice MVA?**

*Invite the person to elaborate (for example environmental factors, support, internal factors)*

1. **What do you see as barriers preventing your from practicing MVA?**

*Invite the person to elaborate (for example environmental factors, support, internal factors)*

1. **Discuss for a moment your level of confidence to practice MVA. What are the elements that impact your confidence to practice MVA?**

*Invite the person to elaborate*

1. **Discuss your personal perceptions and beliefs about the issue of clandestine abortion in the DRC.**
2. **Discuss your thoughts on the role of the midwife regarding clandestine abortion in the DRC. Discuss your thoughts on the role of the midwife regarding clandestine abortion in the DRC.**

**Part Two: Socio-demographic questions**

1. Province of your hospital: 🞎 Kinshasa 🞎 Kongo Central 🞎 other
2. Type of hospital: 🞎 EmONC-Comprehensive 🞎 EmONC Basic
3. Affiliation of hospital: 🞎 private 🞎 public 🞎 Confessional 🞎 other
4. Rural of urban hospital: 🞎 Urban 🞎 rural
5. Professional category: 🞎 Midwife (A1) 🞎 Auxiliary midwife (A3) 🞎 nurse midwife (A2) 🞎 nurse 🞎 other
6. Do you teach ? 🞎 yes 🞎 no
7. How long have you been practicing ?

🞎 Less than 5 years 🞎 6 to 15 years 🞎 more than 15 years

1. Age : 🞎 20-29 years 🞎 30-39 years 🞎 40-49 years 🞎 50-59 years 🞎 60-69 years
2. Man 🞎 Woman 🞎
